# A Feeling for the Cell: Christian de Duve (1917–2013)

**DOI:** 10.1371/journal.pbio.1001671

**Published:** 2013-10-01

**Authors:** Fred Opperdoes

**Affiliations:** de Duve Institute and Université Catholique de Louvain, Brussels, Belgium

## Abstract

Fred Opperdoes pays tribute to a visionary biochemist and cell biologist.

**Figure:**
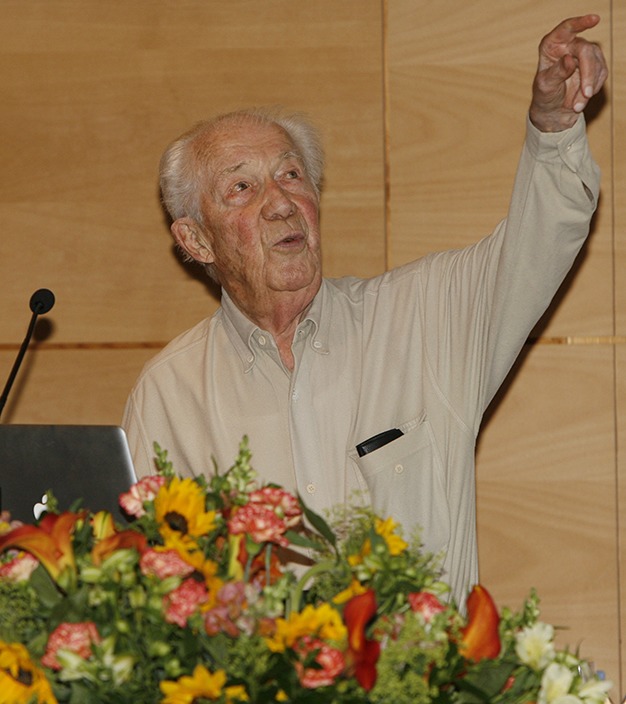
Christian de Duve, age 95, presenting his ideas on the origin of the eukaryotic cell (October 2012). *Image credit: Julien Doornaert*.

Christian de Duve was an internationally renowned cell biologist whose serendipitous observation while investigating the workings of insulin led to groundbreaking insights into the organization of the cell. The observation, which he once described as “essentially irrelevant to the object of our research,” ultimately led him to discover two organelles—the lysosome and the peroxisome—for which he shared the Nobel Prize in Physiology or Medicine in 1974 with Albert Claude and George E. Palade.

Born in 1917 in the United Kingdom to Belgian parents who had fled the devastation of the Western Front during the First World War, de Duve spent his early life in the village of Thames Ditton near London. After the war, in 1920, he and his family returned to Belgium and the young Christian went to school in the Flemish city of Antwerp. He embarked upon his career as a researcher when he enrolled as a medical student at the francophone branch of the Catholic University of Louvain in Belgium (1934–1941). He could speak four languages, a skill that would later help save his life.

de Duve decided to specialize in endocrinology and joined the laboratory of the Belgian physiologist J. P. Bouckaert, where he started his research under the difficult circumstances of the Second World War when facilities and financial support for basic research were very limited. Drafted by the Belgian army, he served as a medical officer in France where he was taken prisoner of war by the Germans. Thanks to his excellent knowledge of German and Flemish, de Duve was able to outwit the enemy and escape back to Belgium, where he immediately returned to his research.

As a young researcher, he initially concentrated on the storage and retrieval of glucose, the body's principal energy source, which is regulated by the pancreatic hormone insulin. In doing so, he discovered that a commercial preparation of insulin happened to be contaminated with another pancreatic hormone, the insulin antagonist glucagon, an insight that led to a better understanding of the mode of action of these two hormones.

After the war, de Duve developed an interest in metabolism to gain a better understanding of the exact mode of action of insulin and glucagon. But his knowledge of biochemistry was still limited, so he decided to widen his horizons. He certainly must have had a gift for selecting the very best laboratories of those days. First, he spent almost a year in Hugo Theorell's laboratory at the Nobel Medical Institute in Stockholm; subsequently, he crossed the ocean and went to Gerty and Carl Cori's laboratory at Washington University in St. Louis where he also had the opportunity to collaborate with Earl Sutherland. Later, all four would become Nobel Prize winners. The Nobel Prize for Physiology or Medicine was received by Carl and Gerti Cori in 1947 for their research on glycogen metabolism, Hugo Theorell in 1951 for his discoveries on oxidation enzymes, and Earl Sutherland in 1971 for the discovery of cyclic AMP.

In 1947, de Duve returned to Belgium to take up the position of professor at the medical school in Louvain and to continue his research on insulin and glucagon. Beginning in 1950, however, he devoted himself more and more to subcellular biochemistry, a new field that he would pioneer together with Albert Claude and George Palade, both in New York. This change of direction was inspired by a chance observation that would change our understanding of the structural and functional organization of the cell: de Duve noticed that the activity of the liver enzyme glucose-6-phosphatase was mostly associated with a sedimentable cell fraction called microsomes, which mainly contained vesicles of the endoplasmic reticulum. (Subsequent analyses established glucose-6-phosphatase as the “marker” enzyme for the endoplasmic reticulum.) Oddly enough, acid phosphatase, which he assayed as a control enzyme during these studies, was latent—it could be activated by various treatments, all of which affected membrane integrity.

Together with his collaborators in Louvain, de Duve concentrated on refining the techniques of differential and isopycnic centrifugation for separating cell constituents, which had been developed by Albert Claude at the Rockefeller Institute (now Rockefeller University) in New York, to isolate the various cellular compartments and to establish their contributions to cell metabolism. Using this approach while working at the Catholic University of Louvain in 1955, de Duve and collaborators reached the conclusion that acid phosphatase and other hydrolases with an acid pH optimum were enclosed in a new organelle, which they proposed to call “lysosome.” Later, together with Alex B. Novikoff of the Albert Einstein College of Medicine in New York, de Duve first saw lysosomes when he made electron micrographs of highly purified cell fractions. These organelles turned out to be loaded with hydrolytic enzymes for the digestion of a multitude of macromolecules taken up by the cell. Thus lysosomes, as we now know, function as the “stomach” of the cell's digestive system. A few years later he discovered a second organelle, which was characterized by the marker enzyme catalase and was loaded mostly with enzymes involved in a dangerous form of oxidation, releasing toxic peroxides, explaining why such reactions should be enclosed within a separate membrane-bound compartment. For this organelle de Duve coined the name peroxisome.

The discovery of these new subcellular organelles paved the way for others to identify lysosomal and peroxisomal diseases. Both are inherited metabolic disorders—mutations or deletions of specific genes that code for either lysosomal or peroxisomal proteins, which results in a dysfunctional cell organelle.

From 1962 onwards, because of his collaborative work with Claude and Palade in New York, de Duve divided his time between the Catholic University of Louvain and the Rockefeller Institute, where he was appointed Andrew W. Mellon professor (1962–1987). In 1968, Belgian politicians decided to split the bilingual Catholic University of Louvain along language lines. The francophone part was moved 30 km south to French-speaking Wallonia, (*l' Université catholique de Louvain* in Louvain-la-Neuve), and de Duve moved to Brussels with the new medical school, which was created at a separate campus from the new francophone university. Immediately after receiving the Nobel Prize, de Duve, together with several of his Belgian colleagues, created a new biomedical institute on the Brussels campus: the International Institute of Cellular and Molecular Pathology (ICP), an institute of translational medicine that was re-named the de Duve Institute on the occasion of his 80th birthday in 1997. de Duve modeled the ICP after the Rockefeller University by giving an absolute priority to scientific excellence and a total freedom of research to its investigators. An experienced administrator, he both ran the institute and acted as fundraiser, a skill he had acquired while in the United States.

In addition to the Nobel Prize, Christian de Duve was the recipient of the Canada Gairdner International Award (1967), the Dr. H. P. Heineken Prize (1973) from the Royal Netherlands Academy of Arts and Sciences, and the E. B. Wilson Award of the American Society for Cell Biology (1989). In 1975, he was elected a foreign associate of the National Academy of Sciences of the United States of America. Eighteen universities around the world awarded him honorary degrees. He was also a member of the Royal Academies of Medicine and the Royal Academy of Sciences, Arts, and Literature of Belgium; the Pontifical Academy of Sciences of the Vatican; the American Academy of Arts and Sciences; the Academy of Sciences of Paris; the Deutsche Akademie der Naturforscher Leopoldina; and the Royal Society of London. In 1989, he was raised to the rank of Viscount by King Baudouin of Belgium.

In the last period of his life, this tireless researcher again shifted his scientific interest, this time from cell biology to the chemical mechanisms that might have led to the origin of life on Earth. A talented writer, he wrote many influential books on this subject as well as on biology and evolution. In total, he wrote more than a dozen books in English and in French, including *A Guided Tour of the Living Cell*; *Blueprint for a Cell: the Nature and Origin of Life*; *Vital Dust: Life as a Cosmic Imperative*; *Life Evolving: Molecules, Mind, and Meaning;* and *Singularities: Landmarks on the Pathways of Life*.

Dr. Christian de Duve remained active until the very end of his life, as this photograph taken in his last year demonstrates. He finished his last book *Sept vies en une: Mémoires d'un Prix Nobel* only a few months before he passed away. When he felt that both his health and strength were rapidly subsiding, he decided to end his life at the age of 95. He chose to die by an act of euthanasia, while surrounded by his children.

Christian de Duve was a brilliant scientist, mentor, and writer. He will be greatly missed by the scientific community.

